# Coping with COVID-19: Exposure to COVID-19 and Negative Impact on Livelihood Predict Elevated Mental Health Problems in Chinese Adults

**DOI:** 10.3390/ijerph17113857

**Published:** 2020-05-29

**Authors:** Jing Guo, Xing Lin Feng, Xiao Hua Wang, Marinus H. van IJzendoorn

**Affiliations:** 1 Department of Health Policy and Management, School of Public Health, Peking University, Beijing 100191, China; 2 School of Social Development and Public Policy, Beijing Normal University, Beijing 100875, China; wxh@bnu.edu.cn; 3 Erasmus School of Social and Behavioural Sciences, Erasmus University Rotterdam, 3000 DR Rotterdam, The Netherlands; marinusvanijzendoorn@gmail.com; 4 School of Psychology, Capital Normal University, Beijing 100048, China; 5 School of Clinical Medicine, University of Cambridge, Cambridge CB2 0SR, UK

**Keywords:** mental health, Post-Traumatic Stress Symptoms (PTSS), depression, insomnia, COVID-19

## Abstract

The COVID-19 pandemic might lead to more mental health problems. However, few studies have examined sleep problems, depression, and posttraumatic symptoms among the general adult population during the COVID-19 outbreak, and little is known about coping behaviors. This survey was conducted online in China from February 1st to February 10th, 2020. Quota sampling was used to recruit 2993 Chinese citizens aged ≥18 years old. Mental health problems were assessed with the Post-Traumatic Stress Disorders (PTSD) Checklist for the Diagnostic and Statistical Manual of Mental Disorders, Fifth Edition (DSM-5), the Center for Epidemiological Studies Depression inventory, and the Pittsburgh Sleep Quality Index. Exposure to COVID-19 was measured with questions about residence at outbreak, personal exposure, media exposure, and impact on livelihood. General coping style was measured by the brief Coping Style Questionnaire (SCSQ). Respondents were also asked 12 additional questions about COVID-19 specific coping behaviors. Direct exposure to COVID-19 instead of the specific location of (temporary) residence within or outside the epicenter (Wuhan) of the pandemic seems important (standardized beta: 0.05, 95% confidence interval (CI): 0.02–0.09). Less mental health problems were also associated with less intense exposure through the media (standardized beta: −0.07, 95% CI: −0.10–−0.03). Perceived negative impact of the pandemic on livelihood showed a large effect size in predicting mental health problems (standardized beta: 0.15, 95% CI: 0.10–0.19). More use of cognitive and prosocial coping behaviors were associated with less mental health problems (standardized beta: −0.30, 95% CI: −0.34–−0.27). Our study suggests that the mental health consequences of the lockdown impact on livelihood should not be underestimated. Building on cognitive coping behaviors reappraisal or cognitive behavioral treatments may be most promising.

## 1. Introduction

The COVID-19 pandemic not only affects physical health, it might also lead to elevated levels of mental health problems such as sleep problems, depressive issues, and posttraumatic stress symptoms [1,2]. The pandemic is, however, a multifaceted and complex type of exposure. Living in the epicenter of the outbreak or having travelled to that center might make a difference in the way the pandemic is experienced. Of course, even more stressful may be the direct experience of COVID-19 infection in relatives, friends or neighbors, or to the person him- or herself. But more distal exposure to the pandemic through the media might also promote mental health problems as individuals may feel intense empathic concern and distress witnessing or constantly hearing about other people’s painful struggles with the coronavirus [3]. Last but not least, the negative impact on livelihood of the pandemic that resulted in an unprecedented lockdown of daily life and economic activities might account for part of the mental health problems in participants whose earning capacities are dwindling [1]. To our knowledge, there are no studies examining these complex types of exposure related to mental health during the COVID-19 outbreak.

In addition, strategies to cope with the strains and stresses implied in the outbreak of the pandemic and social distancing measures in its wake have not been studied extensively. In previous pandemic studies, two types of coping, i.e., general coping style and practical coping behaviors were differentiated. Coping style represents the cognitive and behavioral patterns to manage particular external and/or internal demands appraised as taxing or even exceeding the resources of individuals [4], which has not yet been studied in relation to mental health problems in the context of the COVID-19 pandemic. Practical coping behaviors refer to the ways in which people are likely to behave in a pandemic [5]. Preliminary evidence suggests that careful use of preventive measures such as handwashing and the acquisition of accurate information about COVID-19 predict lower levels of depression and anxiety [6].

What do we know about the pandemic and its mental health effects? In a recent review of the available literature up to the end of March 2020 only four original empirical studies were found [7]. The studies were conducted in China and used self-report measures for anxiety, depression, stress, or sleep problems in a cross-sectional design [6,8,9,10]. The studies found rather substantial stress and trauma-related mental health problems in the general public, sometimes to a higher degree than in front-line workers [8]. Predictably, suffering from COVID-19 symptoms was associated with more depressive feelings and anxiety [6]. Various symptoms of mental health problems seemed to be correlated, for example more anxiety was associated with more sleep problems [9,10].

In the current study we examine mental health problems in Chinese adults during the COVID-19 outbreak. Our main interest was the association between direct versus indirect exposure to the pandemic and level of mental health problems, in particular sleep problems, depressive issues, posttraumatic symptoms, and overall mental health problems. Our first hypothesis concerns location. We expect that participants living in Wuhan or having travelled to this city where the outbreak started would be more vulnerable to the negative consequences of the pandemic than participants outside this epicenter. Our second hypothesis pertains to direct exposure and media exposure to the virus infection, which are expected to predict elevated levels of posttraumatic stress, depressive feelings, and sleeping problems. The third hypothesis is related to impact on livelihood by the pandemic. Due to the lockdown and social distancing measures, the livelihood of many workers may be threatened, and we expect that concerns about the lockdown impact on livelihood predict elevated levels of mental health problems, at least to the same extent as exposure itself does. Lastly, we explore the use of coping behaviors differentiating between problem-focused and emotion-focused coping, without a specific hypothesis as to what kind of coping would be most effective. Testing these hypotheses, we control statistically for several confounders such as previous (mental) health problems, ethnicity, and socio-economic status. Our study is unique in combining differentiated exposures to COVID-19 with mental health problems arising from these exposures and from anxieties about livelihood provoked by the lockdown, in addition to exploring coping styles used during the pandemic.

## 2. Methods

### 2.1. Study Design and Samples

The survey was conducted online from 1–10 February 2020, and the questionnaires were distributed and retrieved through a web-based platform (https://www.wjx.cn/app/survey.aspx). Quota sampling was used to recruit participants. Chinese citizens aged ≥ 18 years old were invited to participate. In total, 2993 participants from 31 provinces in China responded to the survey; 552 students were excluded because of their special status, which resulted in a final *N* = 2441 adults.

To reach more subjects with high exposure to COVID-19 and from somewhat lower social economic strata, we targeted recruitment to six groups that might otherwise have remained underrepresented, namely medical workers, service staff, social service workers, (school and college) teachers, blue-collar workers and farmers, and unemployed individuals and others. The convenience sampling was conducted as followed. First, several key contact persons in these specific groups were selected, for example a chief nurse, class tutor, or company manager. Second, the key contact persons helped us distribute the questionnaires to the subjects through their Wechat group (a very popular Chinese online communication tool). Third, the subjects in each Wechat group were asked to send our questionnaire web link to their friends. This way data were collected from medical workers (*n* = 421, 14.7%), service staff (*n* = 259, 9.1%), social service workers (*n* = 230, 8.0%), (school and college) teachers (*n* = 648, 22.7%), blue-collar workers and farmers (*n* = 388, 13.6%), unemployed individuals and others (*n* = 488, 17.1%). Almost 48% of the respondents were male, and 90.3% of the subjects were between 18–50 years old. More than half of the sample completed at least undergraduate studies, and more than 70% were married. The large majority had a middle to high income and 39% worked in the formal labor market. There were some differences between the participants within Wuhan, in sub-Wuhan, and outside Wuhan (for details, see Table 1).

All participants gave consent after being informed about the aim of the survey and joined the study voluntarily. The study was approved by the Ethics Committee of Peking University Medical Center.

### 2.2. Measures

#### 2.2.1. Outcomes Variables

##### Depression

Depressive symptoms were assessed with the 20-item Center for Epidemiological Studies Depression scale (CESD), which has been widely used to measure depression in the general population [11]. Previous studies demonstrated that this scale has adequate reliability and validity among Chinese respondents [12,13]. Respondents reported the frequency of each type of symptom on a 4-point scale: 0 (rarely or never; less than 1 day), 1 (some of the time; 1–2 days), 2 (a moderate amount of the time; 3–4 days), or 3 (most or all of the time; 5–7 days). The total score ranges from 0 to 60, with a higher score indicating a higher level of depressive symptoms. In this study the Cronbach’s alpha was 0.93. With a cut-off point at 21 [14], respondents were divided into two categories, “depressed” or “not depressed”.

##### Post-Traumatic Stress Symptoms (PTSS)

PTSS were assessed with the self-report Post-Traumatic Stress Symptoms Disorders (PTSD ) Checklist for the Diagnostic and Statistical Manual of Mental Disorders, Fifth Edition (DSM-5), estimating the degree to which individuals had been struggling with DSM-5-related PTSS symptoms in the past month [15]. Respondents answered the 20 items on a 4-point rating scale from 0 (not at all) to 4 (extremely). Items were summed for a total score ranging from 0 to 80, with higher scores indicating higher level of PTSS. Cronbach’s alpha was 0.97. The 20 items were clustered in the following areas: intrusions, avoidance, negative alterations in mood and cognitions, and alterations in reactivity and arousal. The diagnostic criteria of DSM-5 require at least one “intrusion” symptom, one “avoidance” symptom, two “negative alterations in mood and cognitions” symptoms and two “alterations in reactivity and arousal” symptoms, all rated 2 or higher.

##### Insomnia

Sleep problems were assessed using the Pittsburgh Sleep Quality Index (PSQI) [16]. The PSQI consists of 19 items rated from 0 to 3 including estimation of sleep latency, duration, disturbances, and the severity and frequency of other sleep problems. The total PSQI score ranges from 0 to 21 with higher scores indicating worse sleep quality. Cronbach’s alpha was 0.86. With a cut-off point at 7 [17], respondents were divided into two categories, struggling with “insomnia” or “no insomnia”.

##### Mental Health Problems

Because of the high correlations between the scales for Depression, PTSS, and Insomnia, ranging from r = 0.39–0.75, we decided to compute a Principal Component Analysis. A strong first component emerged, with loadings >0.69. The aggregated scale for mental health problems was the sum of scales for PTSS, depressive symptoms, and sleep problems.

#### 2.2.2. Independent Variables

##### Location Wuhan

The question about Wuhan exposure concerned living in or having travelled to Wuhan, with “1” referring to living in or having had a Wuhan travel history, or “0” referring to no Wuhan residence or travel history. Also, two questions were asked about living near Wuhan city, and not living in the vicinity of Wuhan city, with yes or no as possible answers.

##### Media Exposure

Exposure to the COVID-19 pandemic through watching or using the media was answered on a 4-point rating scale for frequency: very frequent, often, some, no exposure).

##### Direct Exposure to COVID-19

Direct exposure to COVID-19 was assessed with a question about possibly having suffered or suffering from COVID-19, or someone in the family, or neighborhood or among friends, with “1” for COVID-19 of self, a member of the family, a friend, someone in the neighborhood, and “0” referring to no exposure).

##### Impact on Livelihood

The respondents were asked to estimate the impact of the pandemic on their livelihood, with four response alternatives (none, some, relatively large, very large impact).

##### Coping Style

It was measured by the simplified Coping Style Questionnaire (SCSQ), developed in China [18]. The SCSQ is a self-report scale which comprises of 20 items with a 5-point rating scale, ranging from 1 (not used) to 5 (used a great deal). The SCSQ consists of two subscales: problem-focused coping and emotion-focused coping. The problem-focused coping category includes twelve items that describe positive cognitive and behavioral strategies to manage distress. The emotion-focused coping category includes eight items that describe negative cognitions and avoiding behavioral activities to manage the problem. This inventory has good internal and test–retest reliability. In the present study, Cronbach’s alpha of the total scale was 0.89 and that for problem-focused coping and emotion-focused coping were 0.85 and 0.93, respectively.

##### Practical Coping Behaviors

Respondents were asked how they were coping with COVID-19. The questions concerned 12 specific coping behaviors, including “tell myself that everything will be better soon”, “reading and watching TV”, “getting more knowledge about COVID-19”, “wearing a mask when going outside”, “staying home and following the social distancing rule”, “disinfecting and deep cleaning”, “crying, being angry, and yelling”, “drinking”, “smoking”, “praying”, “taking more medicine”, and “taking one’s temperature”. The respondents were asked to rate the behaviors from 1 (not used) to 5 (used a great deal).

### 2.3. Covariates

The following covariates were measured. Demographic variables included ethnicity (Han, else), marriage (having no spouse, having a spouse), education (junior high school and below, high school/technical school, junior college, undergraduate, postgraduate and above), and income (low, middle, or high income). Job descriptions included the seven categories mentioned above and categorized into jobs in the formal versus informal sector. Following previous studies [19,20], health-related variables included questions pertaining to prior mental health problems (yes, no), and occurrence of two-week illnesses (yes, no), and prior exposure to potential trauma (experience of a traumatic event in the last year (yes, no)).

### 2.4. Statistical Analyses

The main analyses consisted of multiple regressions on the aggregate outcome of mental health problems in four steps, and in each step the same covariates were used: age, gender, educational level, formal or informal job, married, income, past illness, prior exposure, prior mental health problems. In Model 0 each of the predictors were included separately to estimate their ‘raw’ contribution to the prediction of mental health problems, controlling for the covariates. In Model 1 the three predictors of (potential) exposure (location, media, direct exposure) were included to examine which component would be the most powerful predictor. In Model 2 the perceived impact on livelihood was added, and finally, in Model 3 emotion-focused and problem-focused coping behaviors were included to explore how much variance coping would predict in mental health problems. The standardized beta’s can be compared across models and predictors, lower and upper 95% confidence intervals (CIs) were computed as well as the *p*-values.

In the next series of logistic regression analyses, the odds and their 95% CI and *p*-values for the predictors of the three components of mental health problems were computed, again with the same four models. The components PTSS, depression, and insomnia were dichotomized to differentiate between clinical and nonclinical cases. In a final set of analyses regressions with the 12 coping behaviors as predictors of mental health problems were conducted, controlling for the same set of covariates used in the previous regressions. The Software for Statistics and Data Science (STATA) version 14.0 (StataCorp., College Station, TX, USA) was used to carry out all analyses.

## 3. Results 

### 3.1. Predicting Mental Health Problems

In Table 2 the results of the multiple regressions on the aggregate outcome of mental health problems are presented. The largest variance in mental health problems was explained by coping behaviors, with more use of problem-focused coping behaviors predicting less problems (effect size beta = −0.31), and more use of emotion-focused coping behaviors predicting more problems (effect size beta = 0.50). Furthermore, an important predictor was the perceived impact on livelihood. Larger impacts were associated with more mental health problems and the standardized beta for the respondents feeling the largest impact amounted to a standardized beta of 0.21. Finally, direct or indirect exposure to COVID-19 through location, media, or infected cases predicted statistically significant variance in mental health problems, with Wuhan location, very frequent media exposure, and actual direct exposure to the virus predicting elevated levels of mental health problems. Standardized beta’s ranged from 0.03 to 0.09 (positive or negative), thus considerably smaller effect sizes compared to those found for impact on livelihood or coping. A sensitivity analysis was conducted including formal versus informal job as a predictor of mental health problems instead of its role as a covariate but the beta in Model 3 was a negligible 0.00 (95% CI: −0.04–0.03).

### 3.2. Predicting PTSS, Depression and Insomnia

For predicting PTSS the models showed that exposure through location, media, or direct contact was less important than the impact on livelihood and coping behaviors. More impact on livelihood and more emotion-focused coping were associated with higher risk of clinical PTSS levels, whereas problem-focused coping reduced this risk (see Table 3). This was similar for the prediction of risk for depression, but living in the neighborhood of Wuhan instead of within the city of Wuhan lowered the risk for depression (odds = 0.50), whereas direct exposure added predictive value by elevating the risk of becoming clinically depressed (odds = 1.39, see Table 4). Direct exposure also was associated with elevated risk of insomnia (odds = 1.70, see Table 5).

The models with more predictors included in the same regressions did not make much of a difference compared to Model 0 with only one predictor at a time included (and the covariates of course). Only in Model 3 in which coping was included in the last step the negative beta for living in the neighborhood of Wuhan but not in Wuhan itself was not statistically significant anymore (beta = −0.04). Very frequent media exposure and direct exposure to COVID-19 kept predicting elevated levels of mental health problems. The large effect sizes for impact on livelihood and coping attenuated only slightly from Model 0 to Model 3, and they were still substantial, in particular problem-focused and emotion-focused coping style.

### 3.3. Coping Behaviors and Mental Health Problems

**Figure 1:**
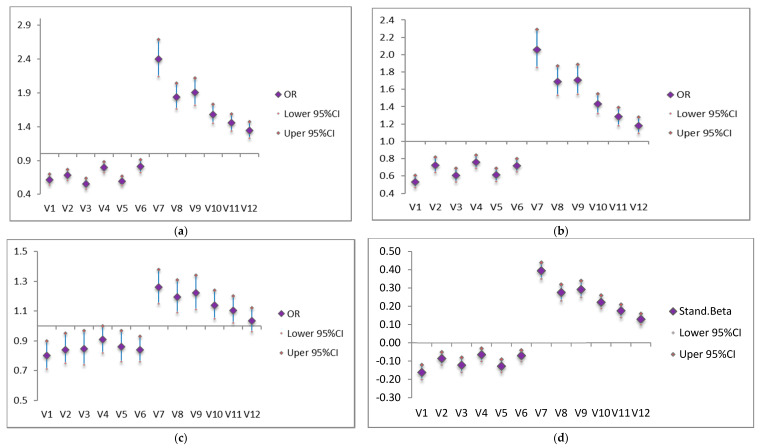
The relationship between coping behavior and Post-Traumatic Stress Symptoms (PTSS), depression, insomnia, mental health problems. (**a**) coping and PTSS, (**b**) coping and depression, (**c**) coping and Insomnia, and (**d**) coping and mental health score. Notes: V1 refers to “tell myself that everything will be better soon”, V2 refers to “reading and watching TV”, V3 refers to “getting more knowledge about COVID-19”, V4 refers to “wearing a mask when going outside”, V5 refers to “staying home and following the social distancing rule”, V6 refers to “disinfecting and deep cleaning”, V7 refers to “crying, being angry, and yelling”, V8 refers to “drinking”, V9 refers to “smoking”, V10 refers to “praying”, V11 refers to “taking more medicine”, and V12 refers to “taking one’s temperature”. These 12 items were asked in a random order in the questionnaire. All confounding variables were controlled in above models. OR, odds ratio. CI confidence interval.

In Figure 1 the practical coping behaviors are presented. More emotion-focused coping behaviors such as “crying, being angry, and yelling”, “drinking”, or “smoking” seemed to be associated with largest risk for mental health problems but more frequently “praying”, “taking more medicine”, or “taking one’s temperature” also elevated this risk albeit to a somewhat lesser extent. Most helpful in decreasing the risk for mental health problems were coping behaviors such as “telling myself that everything will be better soon”, “getting more knowledge about COVID-19”, and “staying home and following the social distancing rule”. To a somewhat lesser extent it seemed also to help when coping with “reading and watching TV”, “wearing a mask when going outside”, and “disinfecting and deep cleaning” were used (see Figure 1a). A similar picture emerged or the association between coping behaviors and PTSS, depression, and insomnia separately (see Figure 1b–d).

## 4. Discussion

Our main findings point at the significant role of direct exposure to COVID-19 instead of the specific location of (temporary) residence within or outside the epicenter of the pandemic. Increased mental health problems were also associated with more intense exposure through the media. Most importantly, in our relatively highly educated and predominantly lower to upper ‘middle-class’ participants the perceived negative impact on livelihood showed the largest effect size in predicting the level of mental health problems. We also examined the effect of coping style and coping behaviors against COVID-19 and found that a problem-focused coping style and positive cognitions and prosocial coping behaviors predicted reduced mental health problems. Compared to Wuhan, we found a lower mental health level among Sub-Wuhan participants. However, this difference disappeared after adjusting for coping. Previous studies on earthquake survivors and on 9/11 world trade center survivors showed that participants who were living closer to the epicenter showed increased mental health issues [21,22]. Our study provides somewhat contrasting evidence for the COVID-19 affected population. Direct exposure, perceived impact on livelihood, and how one was coping with the pandemic seemed more important than the specific location of (temporary) residence within or outside the epicenter (Wuhan) of the pandemic.

For specific mental health problems some different associations were found. Direct exposure to COVID-19 elevated the risk for depression and insomnia but not for clinical PTSS, for which the perceived impact on livelihood seemed more important. Impact on livelihood was also associated with depression but not with insomnia. Direct exposure to COVID-19 involves higher risks for infection and severe respiratory illness, leading to more mental disorders [23], but it is unclear why only the risks of clinical depression and insomnia but not PTSS appeared to be elevated. For PTSS the threat of poverty and the deterioration of economic conditions due to the outbreak seem more important. This threat to livelihood reduces social resources such as access to medical care, education, employment, and well-being for the individual and his or her family, which may cause even greater harm to mental health than the epidemic itself [24].

Our findings demonstrate that coping styles are associated with mental health problems due to COVID-19. A problem-focused coping style seems to relieve individuals’ post-traumatic stress, depression, and insomnia symptoms, and the emotion-focused coping style seems to exacerbate mental health symptoms [25]. Problem-focused coping is a positive strategy that entails some active methods such as finding out several different ways to solve the problem or seeking advices from relatives or friends. Emotion-focused coping tends to emphasize passivity and powerlessness, which enhances anxious and depressed feelings. In line with positive effects of problem-focused coping, we found that practical behaviors such as emphasizing positive cognitions [26] and getting more information about the virus indeed were associated with less mental health problems. In a previous study, positive cognitions about the Severe Acute Respiratory Syndrome (SARS) outbreak were shown to result in less psychological disorders [27]. It also seemed to help when participants tried to cope by following the (pro-)social distancing and hygienic rules. A recent study indicated that personal psychoneuroimmunity prevention measures such as the frequent practice of hand hygiene and wearing face masks could decrease the likelihood that individuals would experience psychiatric symptoms [28]. Through knowledge acquisition and hygienic behaviors individuals actively try to alleviate their fear of uncertainty about the future.

### Implications and Limitations

Some implications may follow from these findings. First and foremost, the mental health consequences of the expectation of a large negative impact on livelihood should not be underestimated. Regardless of location or exposure the economic threats of the pandemic seem to leave a rather strong imprint on mental health. As a worldwide recession has been predicted to follow the current pandemic [29], our findings foreshadow indeed ‘a crashing wave’ not only of immune-system related neuropsychiatric disorders [30], but also of a wide array of stress-related depressive symptomatology without a direct link to deficits of the immune system. The most effective coping behaviors are pointing at cognition and might suggest the potentially promising role of reappraisal interventions [31] or cognitive behavioral treatments [32] in fighting the negative mental health consequences of the pandemic. Also, the positive role of following the rules of social distancing and hygiene may suggest the importance of active, prosocial involvement in the containment or slowing down of the virus infection also for coping with the mental burden of the pandemic. It may induce a collective feeling of empowerment and some control over an otherwise overwhelmingly stressful experience [33].

Some limitations of this study should be mentioned. First, although it is tempting to interpret our findings causally it should be noted that the cross-sectional design without experimental manipulation does not allow for causal conclusions. It is difficult, however, to see how exposure to COVID-19 might be the effect instead of the cause of elevated mental health problems, controlling for pre-existing problems. Nevertheless, the associations with perceived impact on livelihood and with coping behaviors might be (partly) caused by elevated mental health problems, and longitudinal or quasi-experimental studies may throw some light on the causal direction [34]. Second, because we used an internet survey in a large sample it was not possible to include a long series of scales, questions, and items. We relied for example on a simple but clear-cut question about the respondents’ feelings about the impact of the pandemic on their livelihood and we want to emphasize the need for further research on this issue with more elaborated measures. Our findings certainly demonstrate that this is a fruitful path to follow in the near future. Third, generalizability of the results might be restricted in time, geography and sociocultural context. Our data were collected at the beginning of February 2020, a moment in time where the true nature of the pandemic seemed not yet clear to the general public worldwide or even to the experts. Furthermore, the study was conducted in various parts of China but surely did not have worldwide coverage and thus its findings might be (partly) specific to this geographic environment. Lastly, we recruited a Chinese convenience sample for which the nonresponse rate could not be established because of anonymity requirements and in which poor participants from rural areas without internet connections were underrepresented. This underrepresentation of poor participants might have led to an underestimate of the mental health consequences of worries about livelihood issues during and after the lockdown.

## 5. Conclusions

The COVID-19 outbreak in Wuhan was followed by a worldwide pandemic and unprecedented lockdown of many large cities and entire countries. Here we reported on the early mental health sequelae (in the first few weeks of February 2020) of the outbreak in the city Wuhan, the province of Hubei and other provinces in China. We found that direct exposure to COVID-19 and the impact on livelihood are important predictors of mental health problems, and that people found cognitive and prosocial ways to cope with the strains and stresses of the lockdown. We hope that our findings will contribute to the lessons to be learnt about the mental health correlates and consequences of such a pandemic and radical lockdown.

## Figures and Tables

**Table 1:** Descriptive data on social-demographics, exposure, and coping style in the current sample (*N* = 2441).

Characteristics		Within Wuhan		Sub-Wuhan		Outside Wuhan		Total	
		*n*	%	*n*	%	*n*	%	*n*	%
**Gender**	Male	87	41.4	88	46.8	987	48.3	1162	47.6
	Female	123	58.6	100	53.2	1056	51.7	1279	52.4
**Age**	18–25	10	4.8	16	8.5	278	13.6	304	12.5
	26–30	51	24.3	23	12.2	539	26.4	613	25.1
	31–40	69	32.9	73	38.8	747	36.6	889	36.4
	41–50	46	21.9	45	23.9	307	15.0	398	16.3
	≥51	33	15.7	31	16.5	166	8.1	230	9.4
	Missing	1	0.48	0	0	6	0.3	7	0.3
**Education**	Middle school and below	40	19.1	62	32.9	168	8.2	270	11.1
	High school	25	11.9	47	25.0	294	14.4	366	14.9
	Secondary	21	10.0	28	14.9	404	19.8	453	18.6
	Undergraduate	82	39.1	42	22.3	922	45.1	1046	42.9
	Graduate and above	42	20.0	9	4.8	255	12.5	306	12.5
**Married**	No	52	24.8	39	20.7	634	31.1	725	29.7
	Yes	158	75.2	149	79.3	1409	68.9	1716	70.3
**Income**	Poor	21	10.0	23	12.2	218	10.7	262	10.7
	Middle and High	189	90.0	165	87.8	1825	89.3	2179	89.3
**Job**	Formal sector	93	44.3	38	20.2	812	39.8	943	38.6
	Informal sector	117	55.7	150	79.8	1231	60.3	1498	61.4
**Prior** **mental health problems**	No	177	84.3	172	91.5	1752	85.8	2101	86.1
	Yes	33	15.7	16	8.5	291	14.2	340	13.9
**Two-week disease**	Yes	20	9.5	10	5.3	126	6.2	156	6.4
	No	190	90.5	178	94.7	1917	93.8	2285	93.6
**Prior exposure**	Yes	19	9.1	7	3.7	165	8.1	191	7.8
	No	191	90.9	181	96.3	1878	91.9	2250	92.2
**Direct exposure**	Yes	86	40.9	108	57.5	384	18.8	588	24.1
	No	124	59.1	80	42.5	1659	81.2	1853	75.9
**Media exposure**	Very frequent	120	57.1	87	46.3	1196	58.5	1403	57.5
	Often	59	28.1	56	29.8	512	25.1	627	25.7
	Some	14	6.7	26	13.8	165	8.1	205	8.4
	Almost none	17	8.1	19	10.1	170	8.3	206	8.4
**Perceived impact on livelihood**	None	56	26.7	48	25.5	600	29.4	704	28.8
	Some	63	30.0	69	36.7	698	34.2	830	34.0
	Relatively large	43	20.5	33	17.6	437	21.4	513	21.0
	Very large	48	22.9	38	20.2	308	15.1	394	16.1
**PTSS**	Yes	173	82.4	170	90.4	1601	78.4	1944	79.6
	No	37	17.6	18	9.6	442	21.6	497	20.4
**Depression**	Yes	150	71.4	158	84.0	1465	71.7	1773	72.6
	No	60	28.6	30	16.0	578	28.3	668	27.4
**Insomnia**	Yes	53	25.2	34	18.1	415	20.3	502	20.6
	No	157	74.8	154	81.9	1628	79.7	1939	79.4
**Coping style**		**Mean**	**SD**	**Mean**	**SD**	**Mean**	**SD**	**Mean**	**SD**
	Emotion-focused coping	9.9	4.7	8.3	4.8	9.3	5.4	9.3	5.3
	Problem-focused coping	20.8	7.2	19.7	8.1	20	8.7	20.0	8.5

Notes: PTSS: Post-Traumatic Stress Symptoms; SD: standard deviation.

**Table 2:** Multiple linear regressions with exposure and coping style predicting mental health problems.

Independent Variables	Stand.beta	95% CI	Stand.beta	95% CI	Stand.beta	95% CI	Stand.beta	95% CI
	Model 0		Model 1		Model 2		Model 3	
**Location**								
Wuhan (reference)								
Sub-Wuhan	−0.08	−0.13–−0.04 ***	−0.07	−0.12–−0.03 ***	−0.06	−0.11–−0.02 **	−0.04	−0.08–0.00
Outside Wuhan	−0.03	−0.08–0.02	0.00	−0.05–0.05	0.02	−0.04–0.07	0.01	−0.03–0.06
**Media exposure**								
Very frequent (reference)								
Often	−0.09	−0.13–−0.05 ***	−0.09	−0.12–−0.05 ***	−0.07	−0.11–−0.03 ***	−0.05	−0.09–−0.02 **
Some	−0.03	−0.07–0.01	−0.03	−0.07–0.01	−0.03	−0.06–0.01	−0.02	−0.05–0.01
Almost none	−0.07	−0.11–−0.04 ***	−0.07	−0.11–−0.04 ***	−0.07	−0.10–−0.03 ***	−0.07	−0.10–−0.03 ***
**Direct exposure**								
No (reference)								
Yes	0.07	0.04–0.11 ***	0.09	0.05–0.13 ***	0.08	0.04–0.12 ***	0.05	0.02–0.09 **
**Perceived impact on livelihood**								
None (reference)								
Some	0.07	0.03–0.11 ***			0.08	0.04–0.11 ***	0.05	0.02–0.09 **
Relatively large	0.18	0.14–0.23 ***			0.18	0.14–0.22 ***	0.14	0.11–0.18 ***
Very large	0.21	0.16–0.26 ***			0.21	0.16–0.25 ***	0.15	0.10–0.19 ***
**Coping**								
Problem-focused	−0.31	−0.34–−0.27 ***					−0.30	−0.34–−0.27 ***
Emotion-focused	0.50	0.46–0.55 ***					0.47	0.42–0.51 ***

Notes: ** means *p* < 0.01, *** means *p* < 0.001. All variables were standardized before adding into the model. All confounding variables (gender, age, education, married or not, income, job, prior mental health problems, two-week diseases, prior exposure) were controlled in Model 0–Model 3; CI: confidence interval.

**Table 3:** Binary logistic regressions with exposure and coping style predicting PTSS.

Independent Variables	OR	95% CI	OR	95% CI	OR	95% CI	OR	95% CI
	Model 0		Model 1		Model 2		Model 3	
**Location**								
Within Wuhan (reference)								
Sub-Wuhan	0.42	0.23–0.77 **	0.44	0.24–0.81 **	0.47	0.26–0.86	0.54	0.28–1.05
Outside Wuhan	1.12	0.76–1.65	1.27	0.85–1.89	1.38	0.92–2.06	1.43	0.91–2.23
**Media exposure**								
Very frequent (reference)								
Often	0.71	0.55–0.91 **	0.71	0.55–0.92 **	0.75	0.58–0.97	0.78	0.59–1.02
Some	0.83	0.56–1.23	0.85	0.58–1.26	0.82	0.55–1.23	0.86	0.57–1.31
Almost none	0.69	0.46–1.04	0.69	0.45–1.04	0.70	0.46–1.07	0.68	0.44–1.06
**Direct exposure**								
No (reference)								
Yes	1.20	0.95–1.52	1.36	1.06–1.75 *	1.37	1.05–1.77 *	1.21	0.92–1.60
**Perceived impact on livelihood**								
None (reference)								
Some	1.50	1.10–2.04 **			1.53	1.12–2.08 ***	1.49	1.07–2.07
Relatively large	3.09	2.25–4.23 ***			3.09	2.25–4.24 ***	2.95	2.11–4.13 ***
Very large	2.68	1.91–3.76 ***			2.67	1.89–3.76 ***	2.00	1.37–2.93 ***
**Coping**								
Problem-focused	0.91	0.89–0.93 ***					0.91	0.89–0.93 ***
Emotion-focused	1.24	1.20–1.28 ***					1.23	1.19–1.28 ***

Notes: * means *p* < 0.05, ** means *p* < 0.01, *** means *p* < 0.001. All variables were standardized before adding into the model. All confounding variables (gender, age, education, married or not, income, job, prior mental health problems, two-week diseases, prior exposure) were controlled in Model 0–Model 3; OR, odds ratio. CI, confidence interval; PTSS, Post-Traumatic Stress Symptoms.

**Table 4:** Binary logistic regressions with exposure and coping style predicting depression.

Independent Variables	OR	95% CI	OR	95% CI	OR	95% CI	OR	95% CI
	Model 0		Model 1		Model 2		Model 3	
**Location**								
Within Wuhan (reference)								
Sub-Wuhan	0.42	0.25–0.69 ***	0.43	0.26–0.71 **	0.46	0.27–0.75 **	0.50	0.28–0.87 *
Outside Wuhan	0.87	0.62–1.21	1.03	0.73–1.45	1.10	0.78–1.55	1.04	0.70–1.53
**Media exposure**								
Very frequent (reference)								
Often	0.81	0.65–1.02	0.82	0.65–1.03	0.86	0.68–1.08	0.90	0.70–1.16
Some	1.16	0.83–1.63	1.19	0.85–1.68	1.18	0.83–1.66	1.27	0.86–1.87
Almost none	0.80	0.56–1.16	0.80	0.55–1.15	0.83	0.57–1.20	0.79	0.53–1.18
**Direct exposure**								
No (reference)								
Yes	1.42	1.14–1.76 **	1.54	1.23–1.93 ***	1.54	1.22–1.94 ***	1.39	1.08–1.80 *
**Perceived impact on livelihood**								
None (reference)								
Some	1.30	1.00–1.69 *			1.32	1.01–1.72 *	1.25	0.94–1.67
Relatively large	2.50	1.88–3.31 ***			2.46	1.86–3.27 ***	2.39	1.75–3.26 ***
Very large	2.22	1.65–3.00 ***			2.21	1.63–2.99 ***	1.69	1.20–2.37 **
**Coping**								
Positive	0.87	0.85–0.89 ***					0.87	0.85–0.89 ***
Negative	1.30	1.25–1.34 ***					1.29	1.25–1.34 ***

Notes: * means *p* < 0.05, ** means *p* < 0.01, *** means *p* < 0.001. All variables were standardized before adding into the model. All confounding variables (gender, age, education, married or not, income, job, prior mental health problems, two-week diseases, prior exposure) were controlled in Model 0–Model 3; OR, odds ratio. CI, confidence interval.

**Table 5:** Binary logistic regressions with exposure and coping style predicting Insomnia.

Independent Variables	OR	95% CI	OR	95% CI	OR	95% CI	OR	95% CI
	Model 0		Model 1		Model 2		Model 3	
**Location**								
Within Wuhan (reference)								
Sub-Wuhan	0.62	0.38–1.02	0.67	0.40–1.11	0.69	0.42–1.15	0.78	0.46–1.33
Outside Wuhan	0.73	0.52–1.02	0.95	0.66–1.36	0.99	0.68–1.42	0.98	0.67–1.44
**Media exposure**								
Very frequent (reference)								
Often	0.78	0.61–1.00	0.78	0.61–1.01	0.81	0.63–1.04	0.84	0.65–1.09
Some	0.84	0.57–1.23	0.84	0.57–1.23	0.84	0.57–1.24	0.87	0.58–1.30
Almost none	0.77	0.51–1.15	0.76	0.50–1.13	0.77	0.51–1.15	0.78	0.51–1.17
**Direct exposure**								
No (reference)								
Yes	1.79	1.42–2.25 ***	1.84	1.44–2.35 ***	1.84	1.44–2.35 ***	1.70	1.33–2.19 ***
**Perceived impact on livelihood**								
None (reference)								
Some	1.05	0.80–1.38			1.06	0.81–1.40	1.01	0.77–1.34
Relatively large	1.45	1.08–1.96 *			1.44	1.07–1.95 *	1.32	0.98–1.79
Very large	1.60	1.16–2.20 **			1.55	1.12–2.14 **	1.25	0.89–1.75
**Coping**								
Problem-focused	0.95	0.94–0.96 ***					0.95	0.94–0.97 ***
Emotion-focused	1.13	1.10–1.16 ***					1.12	1.10–1.15 ***

Notes: * means *p* < 0.05, ** means *p* < 0.01, *** means *p* < 0.001. All variables were standardized before adding into the model. All confounding variables (gender, age, education, married or not, income, job, prior mental health problems, two-week diseases, prior exposure) were controlled in Model 0–Model 3; OR, odds ratio. CI, confidence interval.
